# shRNA‑mediated knockdown of KNTC1 inhibits non-small-cell lung cancer through regulating PSMB8

**DOI:** 10.1038/s41419-022-05140-w

**Published:** 2022-08-06

**Authors:** Ruijun Liu, Ruili Liu, Zhiyi Guo, Jianghao Ren, Jia Huang, Qingquan Luo, Qiang Tan

**Affiliations:** 1 Shanghai Lung Tumor Clinical Medicine Center, Shanghai Chest Hospital, Shanghai Jiao Tong University, Shanghai, 200030 P. R. China; 2 Department of Stomatology, Ordos central hospital, Ordos, Inner Mongolia 017000 P. R. China

**Keywords:** Non-small-cell lung cancer, Cell migration

## Abstract

In view of the important roles played by Kinetochore proteins in mitosis, we believed that they may contribute to the development and progression of human cancers, which has been reported recently elsewhere. Kinetochore-associated 1 (KNTC1) participates in the segregation of sister chromatids during mitosis, the effects of which on non-small-cell lung cancer (NSCLC) remain unclear. Here, we sought to identify the biological significance of KNTC1 in NSCLC. KNTC1 protein expression in NSCLC tissues was investigated by immunohistochemistry. Lentivirus delivered short hairpin RNA (shRNA) was utilized to establish KNTC1 silence NSCLC cell lines. The effects of KNTC1 depletion on NSCLC cell proliferation, migration, apoptosis, and tumor formation were analyzed by MTT assay, wound-healing assay, transwell assay, flow cytometry assay, and in nude mouse models in vivo. After KNTC1 reduction, NSCLC cell viability, proliferation, migration, and invasion were restrained. A xenograft tumor model was also provided to demonstrate the inhibited tumorigenesis in NSCLC. In addition, the downstream mechanism analysis indicated that KNTC1 depletion was positively associated with PSMB8. The findings of the present study suggested that KNTC1 may have a pivotal role in mediating NSCLC progression and may act as a novel therapeutic target for NSCLC.

## Introduction

Lung cancer is the most commonly diagnosed malignancy type of cancer with extremely high morbidity and mortality [1, 2]. As estimated, approximately one-third of cancer deaths globally are caused by lung cancer [3]. Based on pathological characteristics, lung cancer is primarily divided into small cell lung cancer (SCLC) and non-small-cell lung cancer (NSCLC), especially NSCLC accounts for the majority of lung cancer diagnoses and often presents as an advanced or metastatic disease [4]. Despite multiple treatment methods, such as surgical resection, radiotherapy, chemotherapy, immunotherapy, and targeted therapies [5, 6], the prognosis of NSCLC patients remains poor and the clinical outcomes are still unsatisfactory with a 5-year overall survival rate being <20% [3]. Consequently, there is an urgent need to identify more effective options for NSCLC treatment.

As a proteinaceous structure, kinetochore plays a crucial role in mitosis, which is mainly responsible for attaching chromosomes to the mitotic spindle, coupling force to chromosome movement, and suppressing the anaphase segregation of chromatids [7, 8]. On the other hand, multiple examples demonstrated that kinetochore genes were overexpressed in tumor tissues relative to normal tissue samples [9]. At the same time, these upregulated kinetochore genes in tumors reminded us that this may act as a causal event for tumor occurrence or progression. Kinetochore-associated 1 (KNTC1), an evolutionarily conserved subunit of the kinetochore protein complex, is involved in spindle assembly and chromosomal segregation [10]. However, to the best of our knowledge, there are few previous reports describing the significance of KNTC1 in human malignancies, or in NSCLC in particular.

To this aim, we sought to explore the significance and mechanism of KNTC1 in regulating NSCLC development and progression. Confirming this abundant expression of KNTC1 in NSCLC tissues compared to the corresponding adjacent tissues, we demonstrated that NSCLC cells with downregulated expression of KNTC1 manifested decreased proliferation ability, promoted apoptosis, and reduced migration ability. Consistently, xenografts growth formed by cells with KNTC1 knockdown was obviously suppressed in vivo. Further downstream mechanism revealed that the changes in the biological activity of NSCLC cells caused by KNCT1 knockdown attributed to the downregulation of PSMB8. Therefore, perhaps KNTC1 serves as a promising candidate target for NSCLC gene silencing strategies.

## Materials and methods

### Cell culture

NCI-H1299 and A549 NSCLC cell lines were purchased from Cell Resource Center, Shanghai Academy of Life Sciences, Chinese Academy of Sciences. NCI-H1299 cells were cultured in 1640 medium with 10% FBS. A549 cells were cultured using F12K + 10% FBS medium. All culture medium was changed every 3 days. We performed cell line authentication by short tandem repeat (STR) profiling and tested for mycoplasma contamination (Supplementary materials).

### Immunohistochemistry (IHC)

The formalin-fixed, paraffin-embedded tissue microarray of NSCLC was purchased from Shanghai Outdo Biotech Company (Cat. # HLugA180Su05, China). NSCLC patients’ personal information and pathological data were collected and each patient was informed. The inclusion criteria of the NSCLC samples included in this study were the samples of patients with NSCLC for the survival period. Patients with primary malignancy of other organs prior to diagnosis of NSCLC and who received radiotherapy and chemotherapy were excluded. Our study protocol was approved by the Ethics committee of Shanghai Chest Hospital. The slides were soaked with xylene and washed using 100% alcohol (China National Pharmaceutical Group Co., Ltd, Beijing, China). After, the slides were repaired with 1× EDTA (Beyotime Biotechnology Co., Ltd, Shanghai, China) and blocked with 3% H_2_O_2_ and serum. Next, the primary antibodies and second antibody were used for incubation at 4 °C overnight, and DAB and hematoxylin for staining (Baso DiagnosticsInc., Zhuhai, China). Finally, the slides were sealed with neutral resin (China National Pharmaceutical Group Co., Ltd, Beijing, China) and observed in ×200 or ×400 objective microscopic. The images obtained were analyzed in terms of the IHC scoring.

The IHC score based on the independent identification by three pathologists was used for quantitative analysis. Positive cell score was as 0 (0%), 1 (1–25%), 2 (26–50%), 3 (51–75%), or 4 (76–100%). The staining intensity was scored according to no signal color, light yellow, brownish yellow, and dark brown (from 0 to 3). Specimens were classified into negative (0), positive (1–4), ++ positive (5–8), or +++ positive (9-12), based on the values of positive cell score *the staining intensity. Finally, the high and moderate expression parameters were produced by the median of IHC experimental scores of all tissues. The antibodies used were listed in Supplementary Table 1.

### Lentivirus plasmid construction and transfection

The corresponding RNAi target sequences of KNCT1, S100A10, BAG2, RRP9, DPP3, ZNF655, and PSMB8 were designed by Shanghai Bioscienceres Co., Ltd. (Shanghai, China). The target sequences were inserted into the BR-V-108 vector through the restriction sites at both ends and subsequently transformed into TOP 10 E. coli competent cells (Tiangen, Beijing, China). The positive recombinants were screened by PCR. The EndoFree maxi plasmid kit (Tiangen, Beijing, China) was utilized to extract plasmid, the concentration of which was determined in a spectrophotometer (Thermo_Nanodrop 2000). A three-plasmid BR-V108, BR-V307, BR-V112 co-transfection system was used to collect the 239 T cell supernatant at 48 h and 72 h after transfection and the quality of lentivirus was evaluated. Finally, NCI-H1299 and A549 cells in the logarithmic growth phase were transfected by adding 20 μL 1 × 10^8^ TU/mL lentivirus, culturing in 1640 medium with 10% FBS in a six-well dish with 2 × 10^5^ cells per well. After 72 h-transfection, the cell transfection efficiencies and knockdown efficiencies were evaluated by microscopic fluorescence, qRT-PCR, and western blot.

### Real-time quantitative PCR (qRT-PCR)

Total RNA was extracted according to TRIzol reagent (Sigma, St. Louis, MO, USA). The concentration and quality of the total RNA were determined by Nanodrop 2000/2000C spectrophotometer (Thermo Fisher Scientific, Waltham, MA, USA). Then, the reverse transcription was performed to obtain cDNA by using the Promega M-MLV Kit (Promega Corporation, Madison, Wisconsin, USA). The qRT-PCR system was 10 μL according to the SYBR Green Mastermixs Kit (Vazyme, Nangjing, Jiangsu, China). GAPDH was served as internal reference. The relative expression level was calculated based on the 2^−△△Ct^ method. The primers sequences (5′–3′) were presented in Supplementary Table 2.

### Western blot assay

The total proteins were collected and measured by BCA Protein Assay Kit (HyClone-Pierce, Logan, UT, USA). 20 µg proteins were separated by 10% SDS-PAGE (Invitrogen, Carlsbad, CA, USA) and were transferred onto PVDF membranes. The PVDF membranes were blocked with TBST solution with 5% skim milk at room temperature for 1 h. After that, PVDF membranes were incubated with primary antibodies and second antibodies at room temperature for 2 h. Finally, the complexes were detected using ECL-PLUS/Kit (Amersham, Chicago, IL, USA). Antibodies used in the western blot assay showed in Supplementary Table 1.

### Cell proliferation detection

For Celigo cell counting assay, A549 and NCI-H1299 cells were seeded onto 96-well plates with a cell density of 2500 cells per well. Cells were further cultured in an incubator with 5% CO_2_ at 37 °C. The cell images were taken by Celigo image cytometer (Nexcelom Bioscience, Lawrence, MA, USA) at 24, 48, 72, 96, and 120 h, and a 5-day cell proliferation curve was drawn.

For MTT assay, A549 and NCI-H1299 cells (2,500 cells/well) were seeded onto a 96-well plate. 20 μL MTT solution (5 mg/mL, GeneView, El Monte, CA, USA) was added to each well. 4 h later, 150 μL DMSO was added. Absorbance values at 490 nm were measured using a microplate reader (Tecan, Männedorf, Zürich, Switzerland) after 24, 48, 72, 96, and 120 h of growth, and 570 nm was the reference wavelength. Cell viability ratio was calculated (Cell viability (%) = optical density (OD) treated/OD control × 100%).

### Cell migration and invasion detection

For the Wound-healing assay, A549 and NCI-H1299 cells (4 × 10^4^ cells/well) were seeded onto a 96-well plate. The cell layers in each well were scratched and washed gently 2–3 times with a serum-free medium. Then low-concentration serum medium (0.5% FBS) was added and the cells were incubated in an incubator with 5% CO_2_ at 37 °C. The images were captured by a microscope at 0 h, 8 h, 24 h, and 48 h.

For transwell assay, the upper chamber was in a 24-well plate with 100 μL medium without serum and incubated in an incubator for 1–2 h. A549 and NCI-H1299 cells were collected and resuspended with a low-concentration serum medium and incubated in the upper chamber without a medium (5 × 10^4^ cells/well). In addition, 600 μL medium supplemented with 30% FBS was added to the lower chamber. The upper chamber was transferred to the lower chamber containing 30% FBS medium and incubated in an incubator for 24 h. 400 µL Giemsa was added for cell staining. The migration ability of cells was analyzed.

### Colony formation assay

Lentivirus-transfected A549 and NCI-H1299 cells were collected, digested and resuspended (2,500 cells/mL). For colony formation, 2 mL cell suspension was seeded in a 6-well plate and cultured 8 days, the culture medium was changed every 3 days. Colony photos were collected by fluorescence microscope (Olympus, Tokyo, Japan). Finally, cells were fixed with 4% paraformaldehyde and stained by Giemsa (Dingguo, Shanghai, China) and the number of colonies (>50 cells/colony) was counted.

### Flow cytometry for cell apoptosis and cell cycle

Lentivirus-transfected A549 and NCI-H1299 cells were cultured in 6-well plates (2 mL/well) for 5 days. After that, we collected adherent cells through adding trypsin and complete medium, and transferred them to the centrifuge tube where the supernatant cells were collected. Then, the cell suspension was centrifuged at 1,300 rpm for 5 min. Hereafter, the cells were washed with 4 °C pre-cooled d-Hanks (pH = 7.2~7.4). Next, 10 μL Annexin V-APC (eBioscience, San Diego, CA, USA) was added for staining in the dark for 10–15 min. The cell apoptosis level was measured by using FACSCalibur (BD Biosciences, San Jose, CA, USA).

For cell cycle detection, lentivirus-transfected A549 and NCI-H1299 cells were inoculated in 6 cm dishes (5 mL/well) until the coverage was approximately 80% (The cells did not enter the growth plateau). The supernatant was removed and the cells were washed once with PBS, and then trypsin and complete medium were added to collect the cells. Next, the cell suspension was centrifuged at 1300 rpm for 5 min. The cells were washed with 4 °C pre-cooled PBS, fixed with pre-cooled 70% ethanol for 1 h and stained by adding cell staining solution PI (40× PI, 2 mg/mL: 100× RNase, 10 mg/mL: 1× PBS = 25:10:1000). FACSCalibur (BD Biosciences, San Jose, CA, USA) was used to analyze the change of cell cycle. The percentage of the cells in G1, S, and G2 phase were counted and compared.

### Human Apoptosis Antibody Array analysis

Human Apoptosis Antibody Array (Abcam, Cambridge, MA, USA) was used to detect the changes of apoptosis-related protein expression. After the cells were lysed, the Handling Array membranes were washed and incubated with Wash Buffer II, cell lysates, and Biotin-conjugated Anti-Cytokines overnight at 4 °C. The signals were detected by chemiluminescence imaging system.

### PrimeView human gene expression array

Gene expression in transfected NCI-H1299 cells was detected by RNA screening analysis in Shanghai Bioscienceres, Co., Ltd. (Shanghai, China). Briefly, the total RNA was extracted by the RNeasy kit (Sigma, St. Louis, MO, USA), the quality and integrity of which was determined by Nanodrop 2000 (Thremo Fisher Scientific, Waltham, MA, USA) and Agilent 2100 and Agilent RNA 6000 Nano Kit (Agilent, Santa Clara, CA, USA). According to the manufacturer’s instruction, the RNA sequencing was performed with Affymetrix human GeneChip PrimeView and the outcomes were scanned by Affymetrix Scanner 3000 (Affymetrix, Santa Clara, CA, USA). The statistical significance of raw data was completed by using a Welch t-test with Benjamini-Hochberg FDR (|Fold Change| ≥ 1.5 and *FDR* < 0.05 as significant). Significant difference analysis and functional analysis based on Ingenuity Pathway Analysis (IPA) (Qiagen, Hilden, Germany) was executed, and |*Z* score| > 2 is considered valuable.

### The construction of nude mouse tumor formation model

All animal experiments conformed to the European Parliament Directive (2010/63/EU) and were approved by Ethics committee of Shanghai Chest Hospital. 4-week-old female BALB-c nude mice were purchased from Beijing Weitong Lihua Laboratory Animal Technology Co., Ltd (Beijing, China). 200 μl NCI-H1299 cell suspension (4 × 10^6^ cells) was injected subcutaneously into mice (10 mice/group). During the feeding period, the tumor volume in mice was measured with Vernier caliper. The mice were sacrificed after 34 days. Before sacrificing, 0.7% sodium pentobarbital was injected intraperitoneally at the dosage of 10 mL/g for several min, and the mice were placed under the in vivo imaging system (IVIS Spectrum, Perkin Elmer) to observe the fluorescence. The tumors were removed from the mice and the weight was measured. Tumors were frozen in liquid nitrogen and stored at −80 °C.

### Ki-67 immunostaining

Mice tumor sections were fixed with 4% paraformaldehyde and paraffin-embedded 5 μm sections for IHC staining. The sections were blocked using PBS-H_2_O_2_ with 0.1% Tween 20. Ki-67 antibody was added for incubating at 4 °C overnight and then secondary antibodies were added as described above (Supplementary Table 1). DAB color was developed with diaminobenzene for 10 min and then counterstained with hematoxylin. Stained slides were photographed with a microscopic.

### Statistical analysis

Data were presented as percentages or Means ± SD. SPSS 19.0 software (Chicago, IL, USA) and GraphPad Prism 8.0 (La Jolla, CA, USA) were used. Student’s *t* test was used to analyze the significant differences between two groups, and one-way ANOVA for multiple groups. Spearman rank correlation analysis and Mann–Whitney *U* analysis were used to evaluate the association between KNTC1 expression and characteristics of NSCLC patients. *P* < 0.05 was considered as significant. All the experiments were in triplicate.

## Results

### Increased KNTC1 expression in NSCLC tissues

**Fig. 1:**
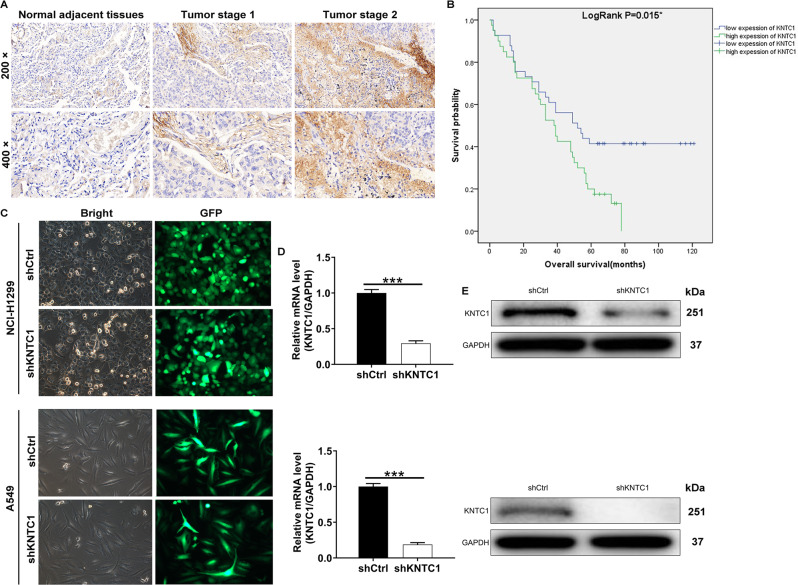
KNTC1 was upregulated in NSCLC tissues. **A** The expression levels of KNTC1 in NSCLC tumor tissues and para-carcinoma tissues were determined by immunohistochemical staining. **B** Kaplan-Meier survival analysis was performed to reveal the relationship between KNTC1 expression and prognosis of NSCLC patients. **C** The transfection efficiencies of shKNTC1 in NSCLC cell lines were evaluated by observing the fluorescence of GFP. Magnification times: ×200. **D** The KNTC1 mRNA expression in NSCLC cell lines after transfection was analyzed by qRT-PCR. **E** The expression of KNTC1 protein in NSCLC cell lines after transfection was detected by western blot. ***P < 0.001.

As the specific regulatory mechanism of KNTC1’s role in NSCLC is unclear, we first explored the expression level of KNTC1 in NSCLC tissues using IHC. In detail, we analyzed KNTC1 expression in 83 NSCLC tissues and 65 normal adjacent tissues, indicating significantly higher KNTC1 level in tumor tissues (*P* < 0.001, Fig. 1A and Table 1). Next, we further examined the correlation between KNTC1 expression and the clinico-pathological characteristics of NSCLC patients. KNTC1 expression and tumor characteristics having correlation data were selected for Spearman correlation analysis. The results showed that KNTC1 was positively correlated with the pathological stage, as shown in Tables 2 and 3. The survivorship analysis (Kaplan-Meier) showed a reduced survival time with the increased KNTC1 expression (Fig. 1B). Taken together, these data might indicate that KNTC1 was extensively linked to the development, progression and prognosis of NSCLC.

**Table 1:** Expression patterns of KNTC1 in NSCLC tissues and para-carcinoma tissues were revealed in immunohistochemistry analysis.

KNTC1 expression	Tumor tissue		Para-carcinoma tissue		*P* value
	Cases	Percentage	Cases	Percentage	
Low	43	51.8%	55	84.6%	<0.001
High	40	48.2%	10	15.4%

**Table 2:** Relationship between KNTC1 expression and tumor characteristics in patients with NSCLC.

Features	No. of patients	KNTC1 expression		*P* value
		low	high	
All patients	83	43	40	
Age (years)				0.916
<63	41	21	20	
≥63	42	22	20	
Gender				0.598
Male	44	24	20	
Female	39	19	20	
Tumor size				0.092
<4 cm	37	23	14	
≥4 cm	46	20	26	
Lymph node positive				0.087
≤1	45	28	17	
>1	35	15	20	
Grade				0.916
I	3	2	1	
II	56	28	28	
III	24	13	11	
Stage				0.003
1	25	19	6	
2	14	8	6	
3	38	15	23	
4	1	0	1	
T Infiltrate				0.229
T1	18	12	6	
T2	44	21	23	
T3	15	8	7	
T4	6	2	4	
lymphatic metastasis (N)				0.078
N0	34	23	11	
N1	15	6	9	
N2	14	5	9	
N3	2	2	0	
Expession of EGFR(Fish)				0.130
negative	63	34	29	
positive	13	4	9

**Table 3:** Relationship between KNTC1 expression and pathological stage in patients with NSCLC.

		KNTC1
Stage	Spearman correlation	0.341
	Signification (double-tailed)	0.002
	*N*	78

### KNTC1 downregulation reduced NSCLC cell proliferation, migration and invasion but promoted apoptosis in vitro

**Fig. 2:**
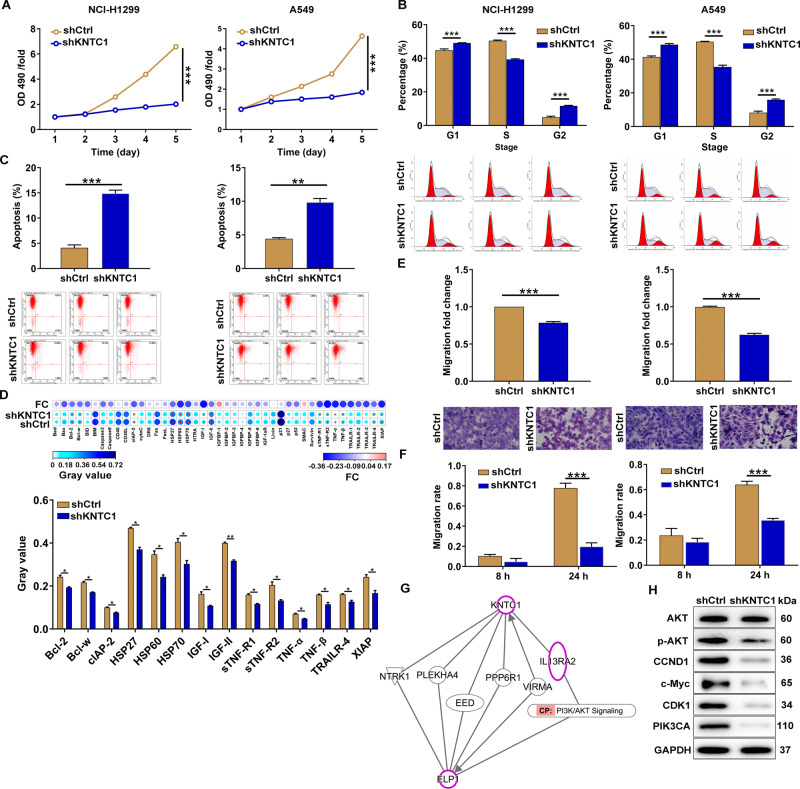
KNTC1 knockdown inhibited NSCLC development in vitro. **A** The cell proliferation rate was evaluated in NSCLC cell lines after transfection by MTT assay. **B** The effects of KNTC1 knockdown on cell cycle were determined by flow cytometry. **C** The effects of KNTC1 knockdown on cell apoptosis were examined by flow cytometry. **D** Human apoptosis antibody array was utilized to illustrate the regulation of the expression of apoptosis-related proteins by KNTC1 knockdown in A549 cells transfected with or without KNTC1. Protein expression was presented in grayscale and visualized by R studio. **E** The migration rate of cells was detected in NSCLC cell lines after transfection by transwell assay. Magnification times: ×200. **F** The migration rate of cells was detected in NSCLC cell lines after transfection by wound-healing assay. **G** The interaction between KNTC1 and AKT pathway was verified via the IPA analysis. **H** The expression of some cancer-associated factors was detected by western blot. Results were presented as mean ± SD. *P < 0.05, **P < 0.01, ***P < 0.001.

Based on the above, we speculated that the decreased expression of KNTC1 might restrain NSCLC development. To verify this, we built up the KNTC1 knockdown models in NSCLC cell lines A549 and NCI-H1299 through transfecting with shKNTC1. In Fig. 1C–E, representative images revealed that KNTC1 levels in A549 and NCI-H1299 cells transfected with shKNTC1 were evidently lower than those transfected with shCtrl, suggesting that KNTC1 knockdown cell model was built up smoothly in both cell lines mentioned earlier. We then explored the effects of KNTC1 knockdown on cell phenotypes through using KNTC1 knockdown cell models. It followed that KNTC1 knockdown markedly cut down the proliferation of NSCLC cells, which was based on the MTT assay (both *P* < 0.001, Fig. 2A). Flow cytometry examination suggested that KNTC1 knockdown arrested cell cycle and induced apoptosis (Fig. 2B, C). Simultaneously, a Human Apoptosis Antibody Array was performed on A549 cells with or without KNTC1 to manifest the regulatory effects of KNTC1 depletion on apoptosis-related proteins, which demonstrated the downregulation of anti-apoptosis proteins including Bcl-2, Bcl-w, clAP-2, HSP27, HSP60, HSP70, IGF-I, IGF-II, sTNF-R1, sTNF-R2, TNF-α, TNF-β, TRAILR-4, and XIAP (*P* < 0.05, Fig. 2D). Furthermore, notably reduced migration and invasion were observed in both KNTC1 knockdown cell models (both *P* < 0.001, Fig. 2E, F). Subsequently, an IPA analysis resulted that KNTC1 could directly affect IL13RA2 in the PI3K/AKT signaling pathway (Fig. 2G). As such, we proposed that the regulation of NSCLC by KNTC1 may be related to the AKT pathway. Next, western blot analysis was performed on A549 cells transfected by shKNTC1 contrasted with shCtrl, indicating the depletion of P-Akt, CCND1, c-Myc, CDK1, and PIK3CA (Fig. 2H). These findings demonstrated that KNTC1 knockdown restrained proliferation, migration and invasion in A549 and NCI-H1299 cells.

### Knockdown of KNTC1 downregulated PSMB8

**Fig. 3:**
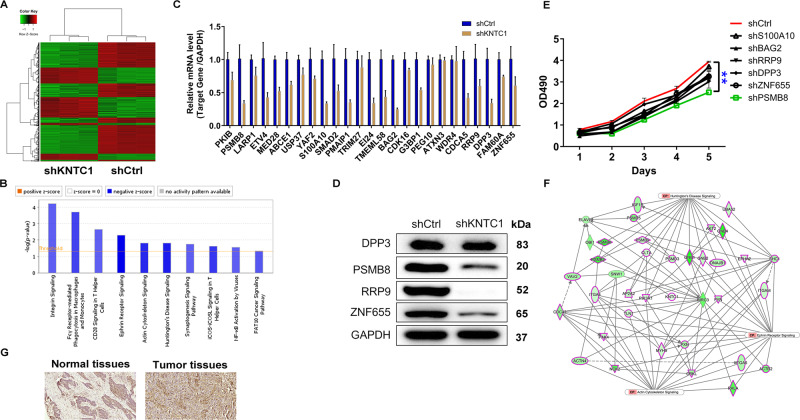
Exploration the underlying mechanism of KNTC1 regulating NSCLC. **A** The heatmap of DEGs identified by RNA-sequencing of NCI-H1299 cells treated with shCtrl (*n* = 3) or shKNTC1 (*n* = 3). **B** The enrichment of the DEGs in canonical signaling pathways was analyzed by the IPA analysis. **C**, **D** The expression of several most significantly differentially expressed genes identified by qPCR (**C**) and western blot (**D**) in NCI-H1299 with shKNTC1. **E** MTT assay was performed to detect the levels of cell proliferation of NCI-H1299 cells after transfecting S100A10, BAG2, RRP9, DPP3, ZNF655 and PSMB8 corresponding lentiviruses. **F** The potential linkage of KNTC1, PSMB8 and Ephrin Receptor Signaling, Huntington’s Disease Signaling and Actin Cytoskeleton Signaling was illustrated via the IPA analysis. **G** The expression levels of PSMB8 in NSCLC tumor tissues and para-carcinoma tissues were determined by immunohistochemical staining. **P < 0.01.

In this regard, we planned to explore the downstream mechanisms that take key functions in KNTC1’s actions towards promoting NSCLC. Of the 970 differentially expressed genes (DEGs) found in shKNTC1 and shCtrl NCI-H1299 cells, 367 was upregulated and 603 was downregulated based on the threshold of absolute fold change ≥1.5 and *FDR* < 0.05 (Fig. 3A). Hereafter, all the DEGs were submitted to IPA software to identify the associated pathways. We found that downregulated genes were enriched in Ephrin Receptor Signaling, Huntington’s Disease Signaling and Actin Cytoskeleton Signaling (Fig. 3B). We next selected 25 downregulated DEGs in these enriched pathway for qRT-PCR detection and four among them for western blot analysis. Especially, among the 25 DEGs, the mRNA and protein expression levels of a few molecules such as S100A10, BAG2, RRP9, DPP3, ZNF655, and PSMB8 were found to be apparently weakened by shKNTC1 in NCI-H1299 cells (Fig. 3C, D). In this way, we constructed lentiviral vectors using corresponding short hairpin RNA for silencing the above-mentioned genes, transfected them into NCI-H1299 cells and detected the levels of cell proliferation. Figure 3E clearly showed that shPSMB8 markedly repressed NCI-H1299 cell proliferation (*P* < 0.01). In addition, the IPA analysis of KNTC1 associated interaction network also illustrated the potential linkage of KNTC1, PSMB8 and those chief pathways identified (Fig. 3F). Additionally, as shown in Fig. 3G, consistent with high expression of KNTC1 in NSCLC tissues, we found a trend towards upregulation of PSMB8. Thus, PSMB8 was considered to be the downstream gene of KNTC1 regulating NSCLC.

### Knockdown of KNTC1 rescued the promotion effects of PSMB8 on cell proliferation and migration and invasion of NSCLC cells

**Fig. 4:**
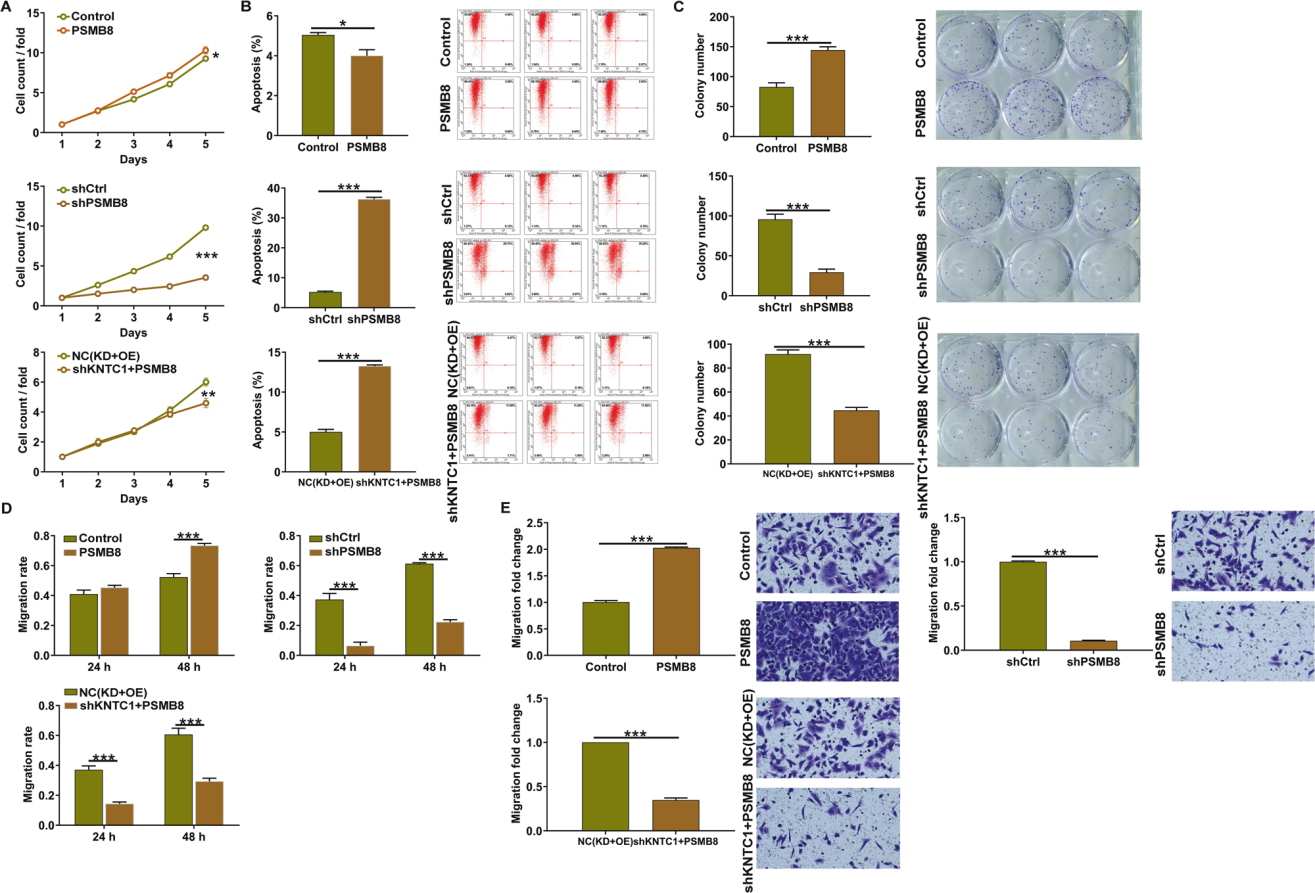
Knockdown of KNTC1 rescued the promotion effects on NSCLC by PSMB8 overexpression. **A** Celigo cell counting assay was employed to show the synergistic effects of KNTC1 and PSMB8 on A549 cell proliferation. **B** The flow cytometry was performed to show the synergistic effects of KNTC1 and PSMB8 on A549 cell apoptosis. **C** Colony formation assay was used to evaluate the ability of A549 cells to form colonies in PSMB8, shPSMB8 and shKNTC1 + PSMB8 groups. **D** The migration rate of cells was detected in PSMB8, shPSMB8 and shKNTC1 + PSMB8 groups by wound-healing assay. **E** The migration rate of cells was detected in PSMB8, shPSMB8, and shKNTC1 + PSMB8 groups by transwell assay. *P < 0.05, **P < 0.01, ***P < 0.001.

With respect to the positive regulatory relationship between KNTC1 knockdown and PSMB8, we next asked the synergistic effects of KNTC1 and PSMB8 on NSCLC cell phenotypes. To solve the query, we established merely downregulating PSMB8, merely over-expressing PSMB8 and simultaneously downregulating KNTC1 and over-expressing PSMB8 cell models through transfecting with shPSMB8, PSMB8 and shKNTC1 + PSMB8 lentiviruses, respectively. The fluorescence inside cells, qRT-PCR assay and western blot assay demonstrated that the three cell models were established well in A549 cells (Supplementary Fig. 1). Besides, cell proliferation was apparently promoted by PSMB8 lentivirus, while was notably cut down by the downregulation of KNTC1 (Fig. 4A). Moreover, flow cytometry assay reflected that the decreased apoptosis level caused by PSMB8 lentivirus could be increased via silencing KNTC1 (Fig. 4B). The colony number was significantly increased in the PSMB8 cell model. By contrast, the colony number in shKNTC1 + PSMB8 cell model was reduced (Fig. 4C). Moreover, the increasing migration and invasion because of overexpressing PSMB8 were notably reduced by KNTC1 knockdown (Fig. 4D, E). As per the results presented so far, we concluded that the promotion effects of PSMB8 on cell proliferation and migration and invasion of NSCLC cells could be rescued via downregulating KNTC1.

### Knockdown of KNTC1 inhibited the growth of NSCLC in vivo

**Fig. 5:**
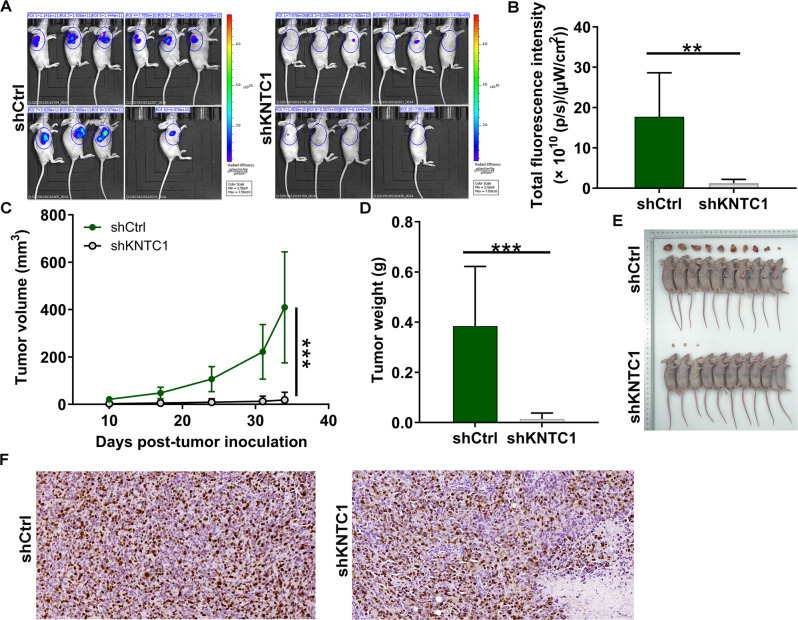
KNTC1 knockdown inhibited NSCLC tumor growth in vivo. **A** A nude mice model of KNTC1 knockdown was constructed. **B** The fluorescence intensity was obtained through injection of d-Luciferase before sacrificing the mice. **C** The volume of tumors was tested from feeding to sacrifice. **D** The weight of tumors was measured after sacrificing mice. **E** The photograph of tumors was taken after removing tumors. **F** The value of Ki-67 was detected by IHC in tumor sections. **P < 0.01, ***P < 0.001.

To further test the roles of KNTC1 knockdown in vivo, the xenograft models in nude mice were established (Fig. 5A). Such decrease in fluorescence was considered to be impaired tumor growth in shKNTC1 treatment (*P* < 0.01, Fig. 5B). Besides, the tumor volume was reduced on the 10th, 17th, 24th, 31th, and 34th day after shKNTC1 transfected NCI-H1299 cells inoculation (Fig. 5C, all *P* < 0.001). Besides, 34 days after the injection of the cell suspension, the mice were killed and the tumor weight was examined. Figure 5D, E manifested that tumor weight was apparently decreased by KNTC1 knockdown (*P* < 0.001). Finally, the expression pattern of Ki-67 related to proliferation was diminished (Fig. 5F). The above suggested the inhibitory roles of KNTC1 knockdown in NSCLC growth in vivo.

## Discussion

Chromosome separation and cell division are both critical biological processes, in which there are numerous evolutionarily conserved protein complexes. Growing evidences reported that these proteins were overexpressed in human malignancies, and some of them even were identified as oncogenes [10]. For example, KNTC2 was reported to be upregulated in various types of cancers, including gastric cancer, colorectal cancer, pancreatic cancer, hepatocellular carcinoma, breast cancer and NSCLC [11–16]. Furthermore, silencing KNTC2 could suppress cell proliferation and induce apoptosis of these above tumor cells [14, 15]. Additionally, targeted knockdown kinetochore scaffold 1 in tumor cells resulted in the obvious inhibition on cell phenotypes [17]. KNTC1 plays a key role in mitosis and ensures proper separation of chromosomes during cell division [10]. Published literature showed that KNTC1 was widely expressed in hepatocellular carcinoma tissues and was related to poor prognosis [18]. In addition, KNTC1 was reported to correlate with esophageal squamous cell carcinoma, and downregulation of KNTC1 expression inhibited cell viability and induced cell apoptosis in ESCC cell lines [19]. These studies demonstrated that kinetochore proteins may work as potential biomarkers for the early diagnosis of cancer, and the characterization of the role of kinetochore proteins may further contribute to the development of novel personalized treatments for human malignancies.

In our current study, we found the upregulation of KNTC1 in NSCLC tumor tissues compared with normal tissues, which was in line with the previously reported tumor promotion effects of KNTC1. Also, high KNTC1 expression was significantly correlated with pathological stage of patients with NSCLC. Subsequently, the loss-of-function experiments revealed that KNTC1 knockdown blocked NSCLC development in vitro and in vivo. As the next step, KNTC1 knockdown caused some cancer-associated factors P-Akt, CCND1, c-Myc, CDK1 and PIK3CA depletion. More importantly, the mechanisms underlying KNTC1‑mediated promotion of NSCLC tumorigenesis was explored, identifying PSMB8 as a downstream target of KNTC1.

Proteasome subunit beta type-8 is a member of the 17 essential subunits for the complete assembly of the 20 S proteasome complex [20]. The 20 S proteasome is composed of seven α-type structural subunits and seven β-type structural subunits, which act as gate structures and form proteolytic domains, respectively [20, 21]. PSMB8, as a member of the immunoproteasome, has higher chymotrypsin-like activity than the standard proteasome [22, 23]. The immunoproteasome could facilitate antigen presentation for CD8+ T-cell responses. Previous research reported that PSMB8, PSMB9 and PSMB10 expressed at significantly abundant levels in dendritic cells, monocytes, and CD8+ T-cells in idiopathic inflammatory myopathies, which correlated highest with STAT1, IRF1 and IFN-γ expression [24]. On the other hand, PSMB8 still involves immune suppressive signaling pathways [25]. Specifically, the activation of STAT3 activates the DNA methyltransferase 1, which methylates the promoters and, therefore, silences the expression of IRFs, human leukocyte antigen molecules, and subunits of the immunoproteasome complex (PSMB8 and PSMB9), thereby reducing antigen presentation [26]. As such, many studies have focused on the relationship between PSMB8 and human cancers. It was reported that PSMB8 overexpression was related to gastric cancer development and progression, especially aspects correlated with the depth of tumor invasion and lymph node metastasis [27]. In glioblastoma, PSMB8 inhibition enhanced apoptosis and suppressed migration and invasion through PI3K/AKT regulation [28]. We found that in NSCLC, PSMB8 was overexpressed and PSMB8 upregulation promoted cell proliferation, migration and invasion of NSCLC cells.

Thus, in conclusion, we found the upregulated expression of KNTC1 and PSMB8 in tumor tissues of NSCLC. Both KNTC1 and PSMB8 could act as tumor promotor in the development and progression of NSCLC through promoting cell proliferation, colony formation, cell migration and suppressing cell apoptosis. More importantly, experimental evidence demonstrated that KNTC1 may regulate NSCLC through its downstream target PSMB8. Therefore, all the results identified KNTC1 as a possible therapeutic target for NSCLC treatment. Developing KNTC1 inhibitors or combining it with potential chemical agents such as paclitaxel may be a promising treatment strategy for advanced NSCLC, and may also be used to against the resistance of NSCLC patients to paclitaxel.

## Supplementary information

Supplementary figure legends

Figure S1

Supplementary Table 1 and Table 2

Supplementary materials-A549 STR profiling

Supplementary materials-NCI-H1299 STR profiling
