# Supplementary material for: shRNA‑mediated knockdown of KNTC1 inhibits non-small-cell lung cancer through regulating PSMB8
Source: Cell Death Dis. 2022 Aug 6;13(8):685. doi: 10.1038/s41419-022-05140-w (PMC9357013; doi:10.1038/s41419-022-05140-w)
Supplement: Supplementary file 1 — Supplementary figure legends [file 41419_2022_5140_MOESM1_ESM.docx]

**Figure S1.** The transfection efficiencies of PSMB8, shPSMB8 and shKNTC1+ PSMB8 in A549 cells were evaluated through observing the fluorescence inside cells, qRT-PCR detection and western blot analysis. Magniﬁcation times: 200×. * *P* < 0.05.
