# Supplementary figures and images for: shRNA‑mediated knockdown of KNTC1 inhibits non-small-cell lung cancer through regulating PSMB8
Source: Cell Death Dis. 2022 Aug 6;13(8):685. doi: 10.1038/s41419-022-05140-w (PMC9357013; doi:10.1038/s41419-022-05140-w)

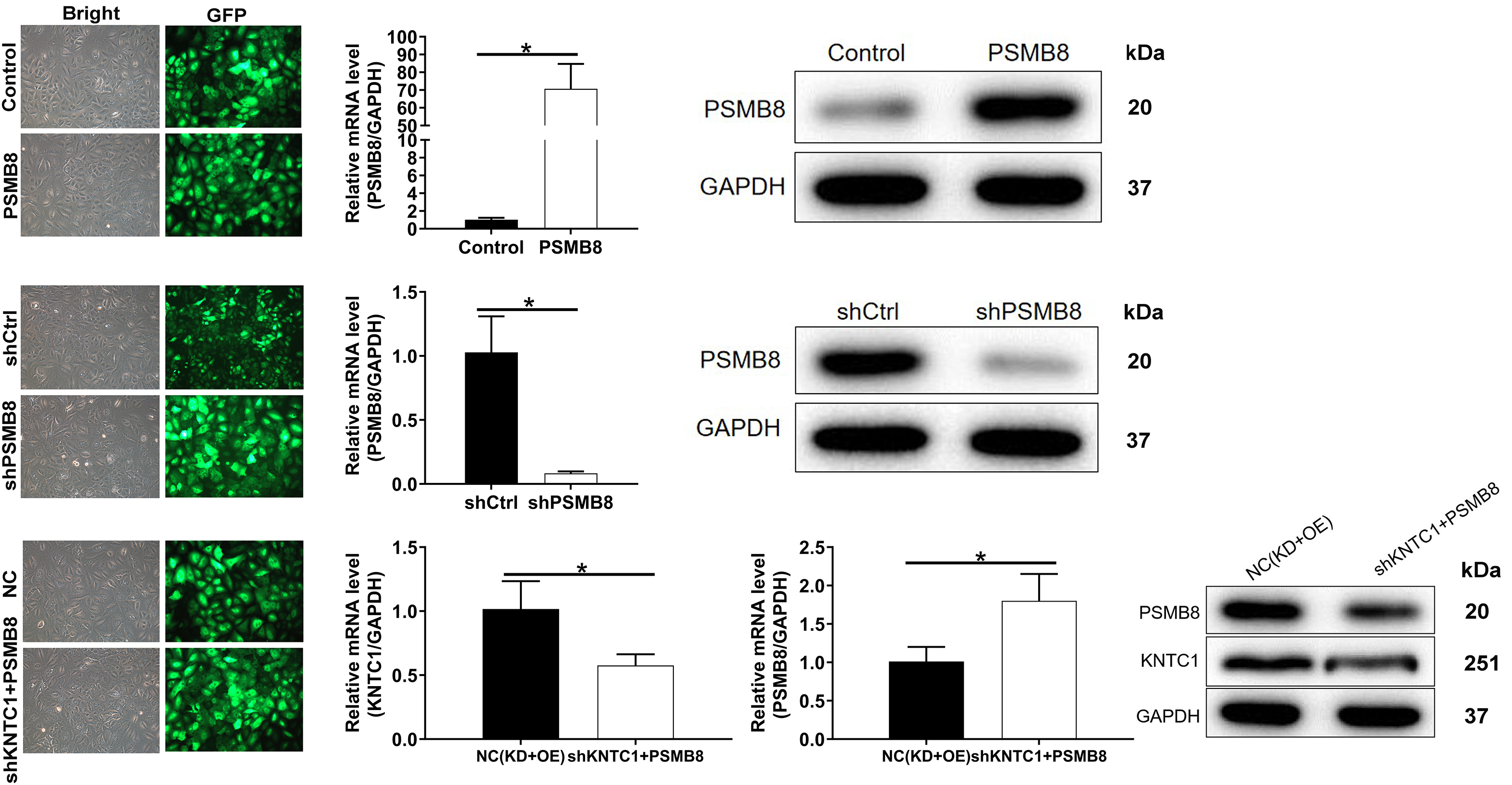

Supplement: Supplementary file 2 — Figure S1 [file 41419_2022_5140_MOESM2_ESM.tif]

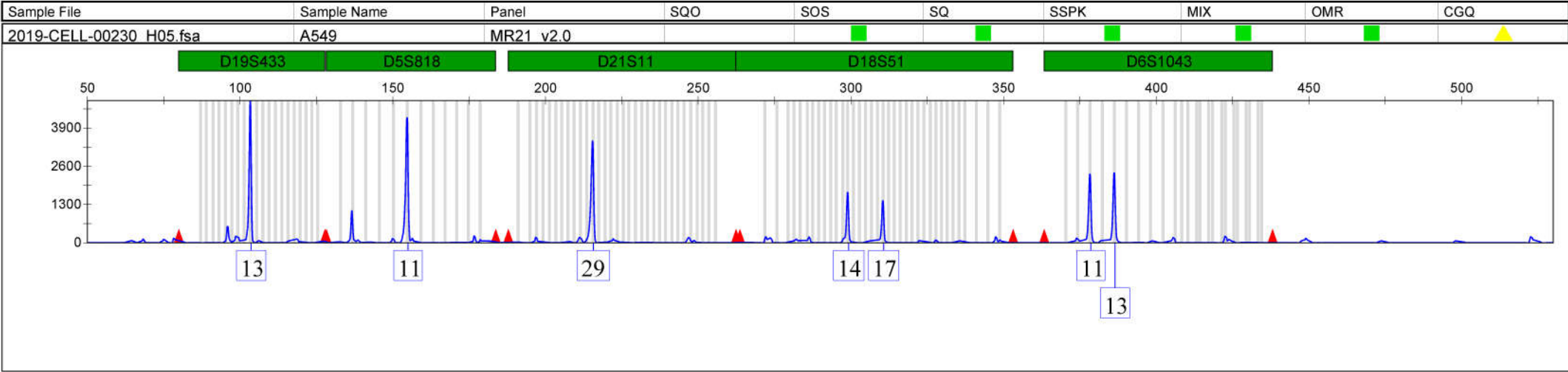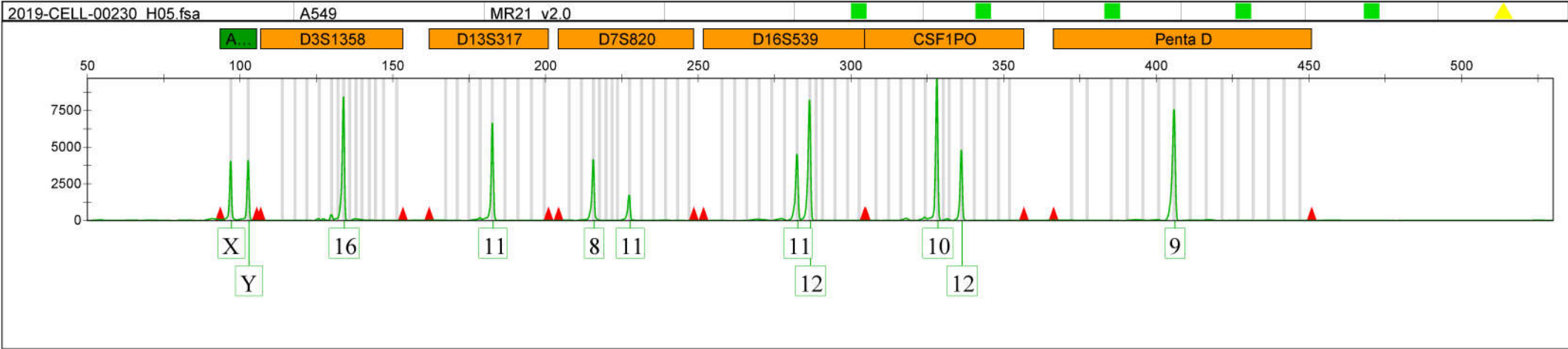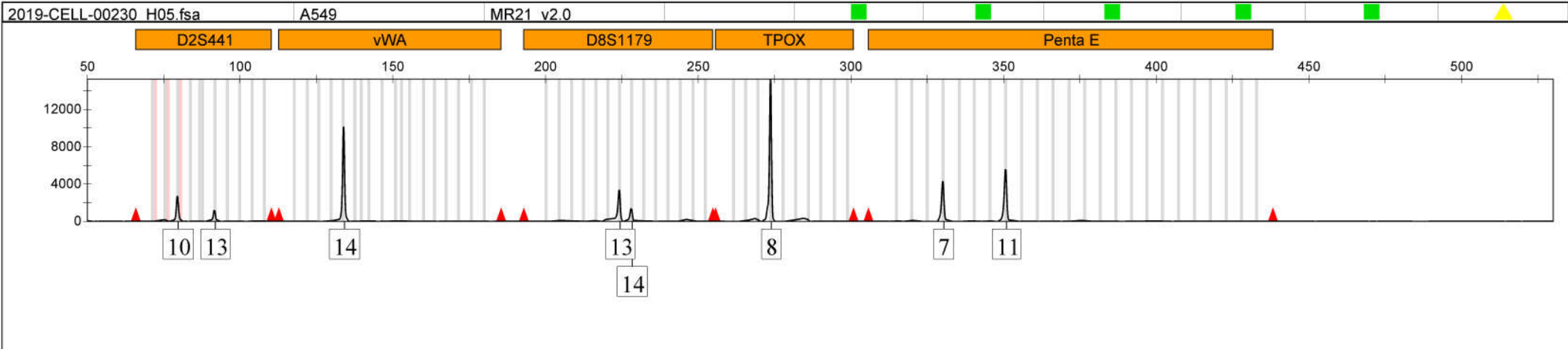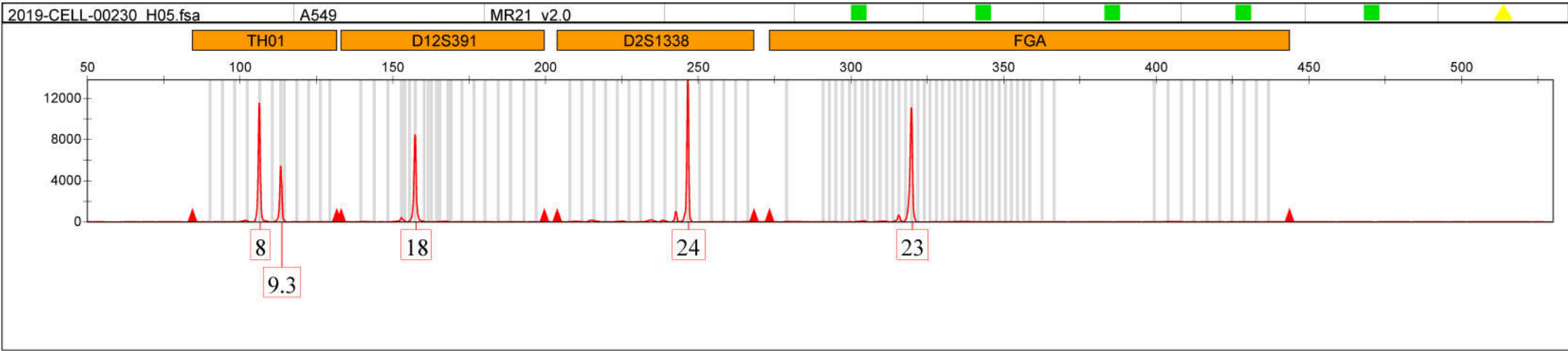

Supplement: Supplementary file 4 — Supplementary materials-A549 STR profiling [file 41419_2022_5140_MOESM4_ESM.pdf]
