# Supplementary material for: shRNA‑mediated knockdown of KNTC1 inhibits non-small-cell lung cancer through regulating PSMB8
Source: Cell Death Dis. 2022 Aug 6;13(8):685. doi: 10.1038/s41419-022-05140-w (PMC9357013; doi:10.1038/s41419-022-05140-w)
Supplement: Supplementary file 3 — Supplementary Table 1 and Table 2 [file 41419_2022_5140_MOESM3_ESM.docx]

Supplementary Table 1. Antibodies used in western blotting and IHC.

| Primary antibodies | Dilution in WB | Source species | Company | Catalog No. |
| --- | --- | --- | --- | --- |
| KNTC1 | 1:1000 | Rabbit | biorbyt | orb101601 |
| Akt | 1:1000 | Rabbit | CST | 4685 |
| P-Akt | 1:500 | Rabbit | R&D | AF887-sp |
| CCND1 | 1:1000 | Rabbit | CST | 2978 |
| c-Myc | 1:1000 | Rabbit | CST | 13987T |
| CDK1 | 1:3000 | Rabbit | abcam | ab133327 |
| PIK3CA | 1:1000 | Rabbit | abcam | ab40776 |
| DPP3 | 1:2000 | Rabbit | Invitrogen | PA5-35038 |
| PSMB8 | 1:1000 | Rabbit | abcam | ab3329 |
| RRP9 | 1:1000 | Mouse | Abnova | H00009136-M01 |
| ZNF655 | 1:1000 | Rabbit | Invitrogen | PA5-56183 |
| GAPDH | 1:3000 | Rabbit | Bioworld | AP0063 |
|  |  |  |  |  |
|  |  |  |  |  |
| Secondary antibodies | Dilution |  | Company | Catalog No. |
| Goat Anti-Rabbit | 1:3000 |  | Beyotime | A0208 |
| Goat Anti-Mouse | 1:3000 |  | Beyotime | A0216 |

| Primary antibodies | Dilution in IHC | Source species | Company | Catalog No. |
| --- | --- | --- | --- | --- |
| KNTC1 | 1:100 | Rabbit | biorbyt | orb101601 |
| PSMB8  Ki67 | 1:200  1:200 | Rabbit  Rabbit | abcam  abcam | ab3329  Ab16667 |
|  |  |  |  |  |
|  |  |  |  |  |
| Secondary antibody | Dilution |  | Company | Catalog No. |
| Goat Anti-Rabbit | 1:400 |  | abcam | ab6721 |

Supplementary Table 2. Primers used in qRT-PCR.

| **Gene** | **Forward primer sequence (5′-3′)** | | **Reverse primer sequence (5′-3′)** |
| --- | --- | --- | --- |
| KNTC1 | TGAAACGCTGCTCCACAAC | | TGCTCGTCAGTAAAGGAACCAT |
| PKIB | ATGCCTTACCAGACATCCAGAG | | TCTTCATTTTGAGGCTTTTCCA |
| PSMB8 | ACAGTGGCTATCGGCCTAATC | | CTTTCACCCAACCATCTTCCT |
| LARP1 | TCTGTTTGACGAGGAGATGGAGC | | GGTGTCTGGGTGACAATGAGGAT |
| ETV4 | CTGGACATTTGCCACTCCTTCACA | | CATACAGGGGATCATGGTATTCTTGC |
| MED28 | AGGCGCTCCTAGACCTTCCA | | CTGATCGGTGCCATTGACATAG |
| ABCE1 | TATCCCTCGTCCAGGTGAAG | | CAGGAGGATCATCGTACTTTC |
| USP37 | GACTGTAGCAGGAAGTGGAA | | TCAATTCTGAGCCAGTTGAT |
| YAF2 | CAGCAGTTTGTGCCTCCTAC | | TTTCAATCTTGGCCTGGTTT |
| S100A10 | CTTCCAGAGCTTCTTTTCCC | | CTACTTCTTTCCCTTCTGCTTC |
| SMAD2 | TCCATCTTGCCATTCACGC | | CCACTTTTCTTCCTGCCCATT |
| PMAIP1 | AGCAGAGCTGGAAGTCGAGTG | | TCCTGAGCAGAAGAGTTTGGAT |
| TRIM27 | AGGGAGAAGATTGTTTGGGAGTT | | TGGCACCATTGATGCTATTGTAG |
| EI24 | TGGAGATGTTTGGTCGTGGCT | | CCTCCCTGATACCTCAAATGC |
| TMEM158 | TGCTTCGTGCTGTAGTTATCGTTAGTTCCT | | TGCGATCCCACCCTCAGTCCAA |
| BAG2 | CAGCAGCAAGAATCCCTAAAGC | | CATGTGGCACCTCAGATGAAC |
| CDK16 | AGATCAGACTGGAACATGAAGAGG | | TCAAAGACAAGGGTGAGGGAC |
| G3BP1 | CTGAAATCCAAGAGGAAAAGCC | | GTCACAGATGCCCAAGAAAATG |
| PEG10 | GCCAAACGCAAGATCAGACG | | GCCCTCGTGGTACTGGTCAAT |
| ATXN3 | CATAAAACAGACCTGGAACGAGTG | | CCATGTCAATTTCTTGGCGACT |
| WDR4 | TGACCGCTTCATCCTCACTGCC | | ACCACGGAGATACGGCTCACAAAC |
| CDCA5 | CCGAGCATCCTCCCTGAAAT | CATGGGCCACGATCCTCTTTA | |
| RRP9 | CAGCATCATTAAGTGGAGCGTG | TACTTGCCGTCGGAGGAGAT | |
| DPP3 | TGAGTGCCAAGTTTGAGCG | AGCGAAGGTGAGAACATCCAG | |
| FAM60A | CAAGCCAAAGATGTACCGAAGT | CATTGCAGATGTCTCCTGAACGA | |
| ZNF655 | AAATACCAGCCCAGGAAGCA | AAACTGAGGCTCTGGGGACA | |
| GAPDH | TGACTTCAACAGCGACACCCA | CACCCTGTTGCTGTAGCCAAA | |
