# Supplementary material for: shRNA‑mediated knockdown of KNTC1 inhibits non-small-cell lung cancer through regulating PSMB8
Source: Cell Death Dis. 2022 Aug 6;13(8):685. doi: 10.1038/s41419-022-05140-w (PMC9357013; doi:10.1038/s41419-022-05140-w)

CellLineAuthentication-11

Sample File	Sample Name	Panel	OS	SQ
29_E04_CellLineAuthentication-0309.fsa	H1299	STR Profile 1-human		

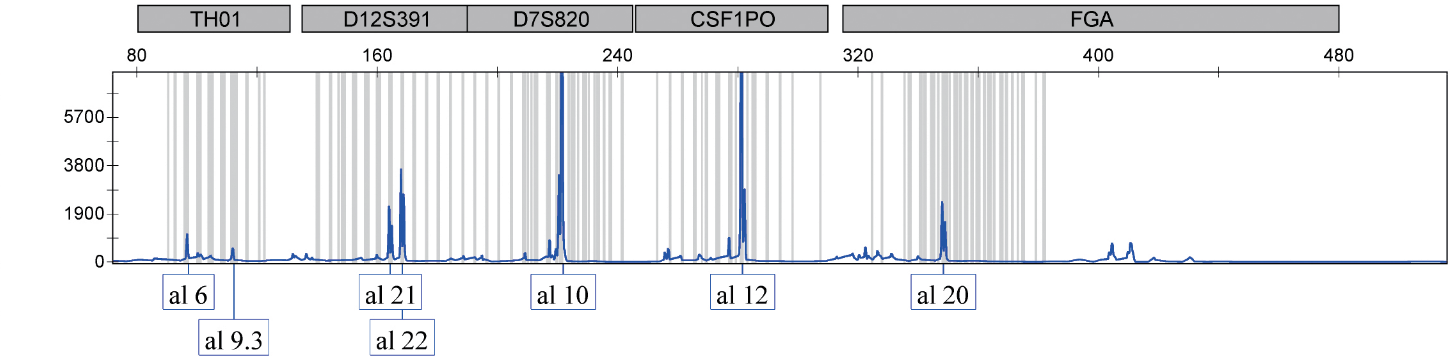

Sample File	Sample Name	Panel	OS	SQ
29_E04_CellLineAuthentication-0309.fsa	H1299	STR Profile 1-human		

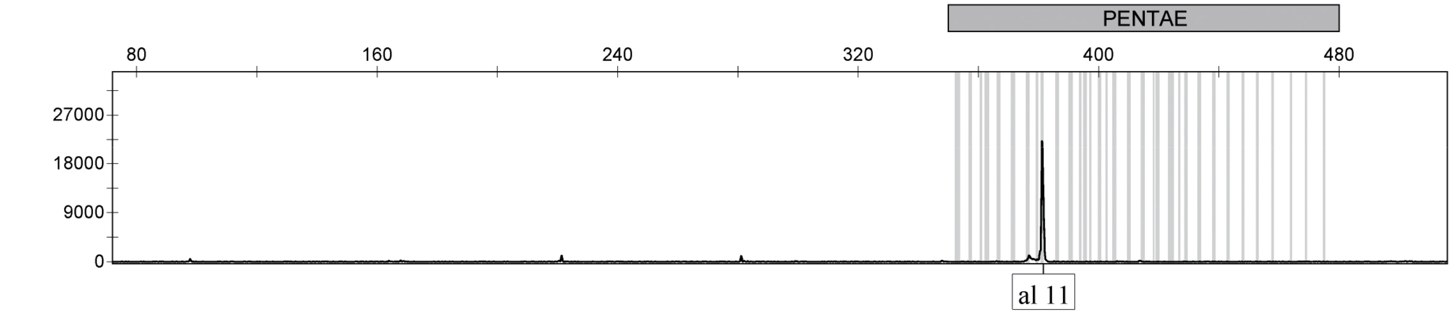

Sample File	Sample Name	Panel	OS	SQ
45_E06_CellLineAuthentication-0309.fsa	H1299	STR Profile 2-human		

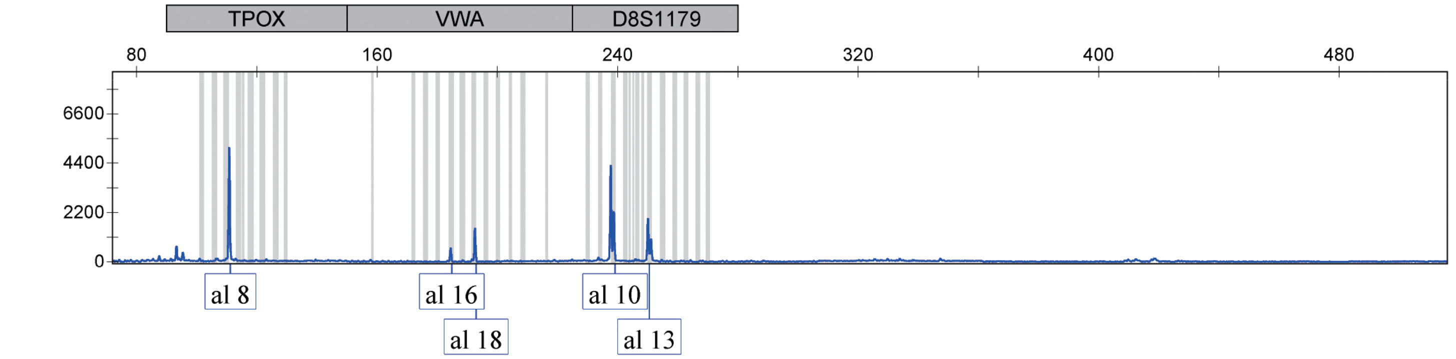

GeneMapper 4.0

CellLineAuthentication-11

Sample File	Sample Name	Panel	OS	SQ
45_E06_CellLineAuthentication-0309.fsa	H1299	STR Profile 2-human		

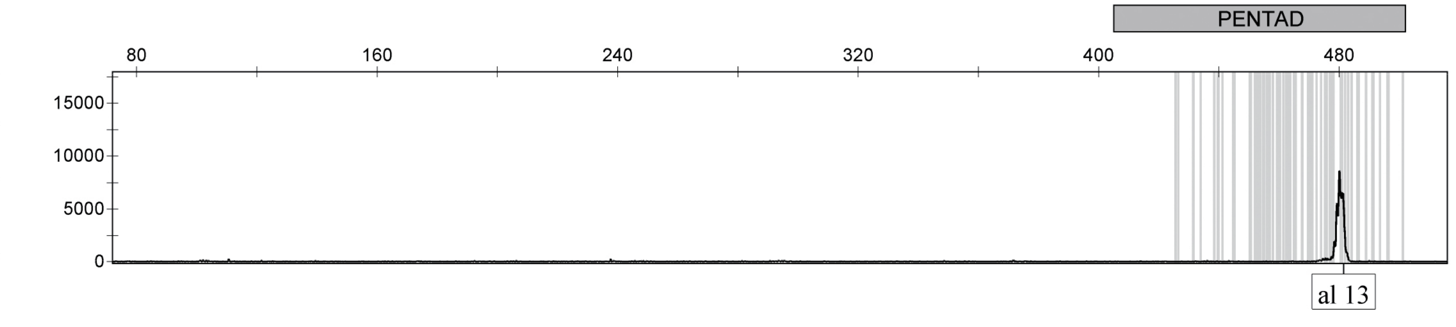

Sample File	Sample Name	Panel	OS	SQ
69_E09_CellLineAuthentication-0309.fsa	H1299	STR Profile 3-human		

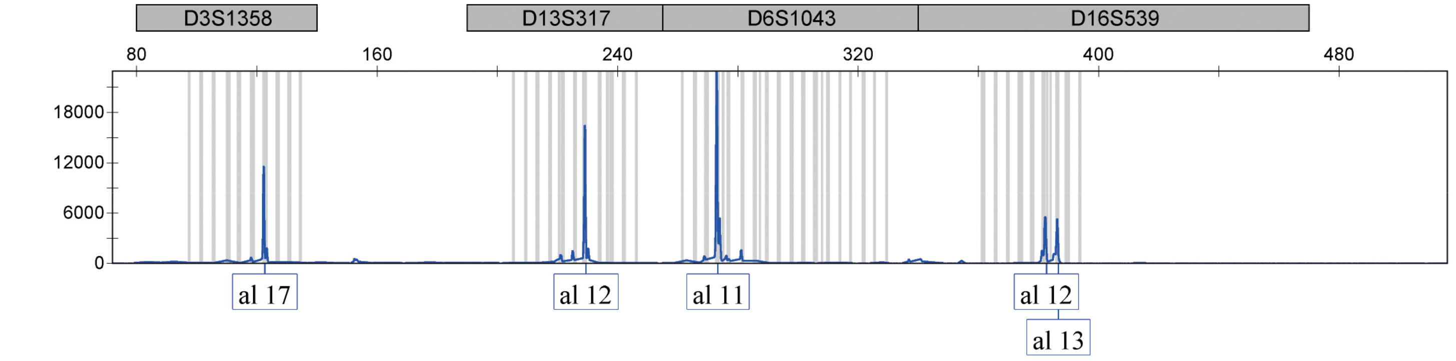

Sample File	Sample Name	Panel	OS	SQ
69_E09_CellLineAuthentication-0309.fsa	H1299	STR Profile 3-human		

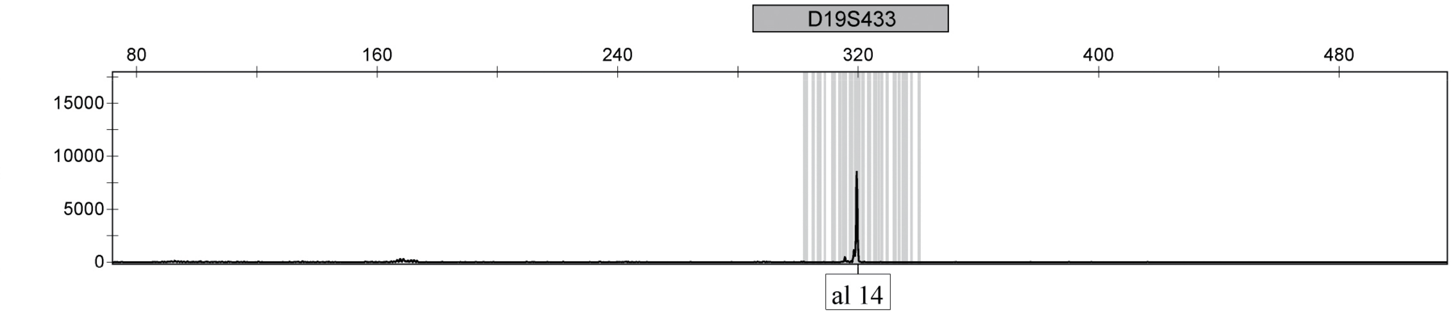

Sample File	Sample Name	Panel	OS	SQ
53_E07_CellLineAuthentication-0309.fsa	H1299	STR Profile 4-human		

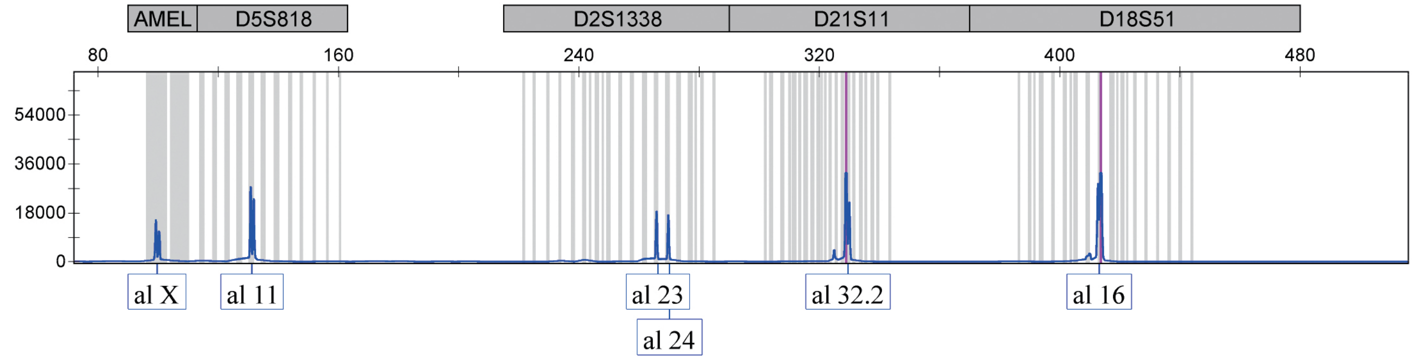

53_E07_CellLineAuthentication-0309.fsa	H1299	STR Profile 4-human		

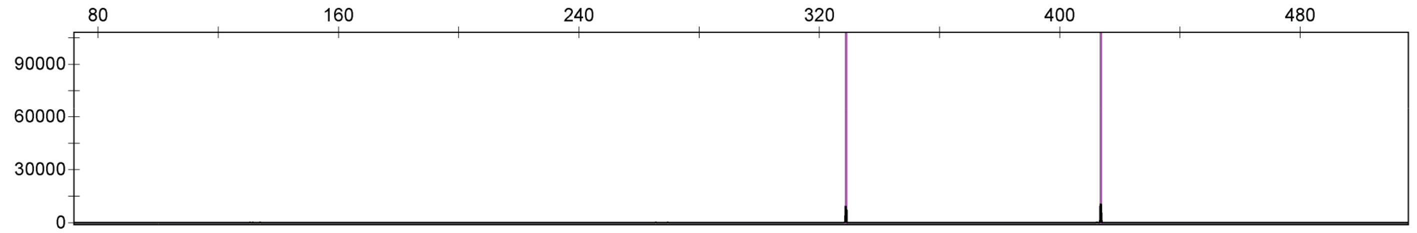

Applied Biosystems
GeneMapper 4.0

CellLineAuthentication-11

Sample File	Sample Name	Panel	OS	SQ
45_E06_CellLineAuthentication-0309.fsa	H1299	STR Profile 2-human		

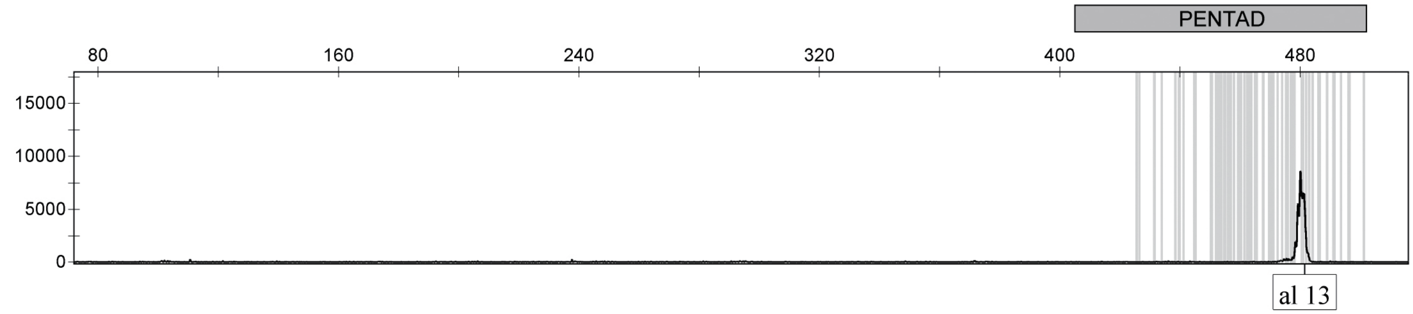

Sample File	Sample Name	Panel	OS	SQ
69_E09_CellLineAuthentication-0309.fsa	H1299	STR Profile 3-human		

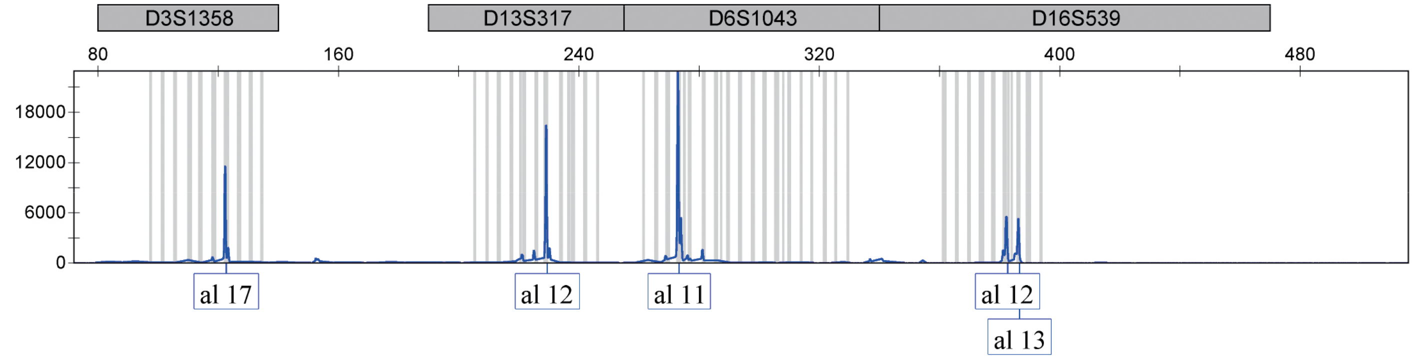

Sample File	Sample Name	Panel	OS	SQ
69_E09_CellLineAuthentication-0309.fsa	H1299	STR Profile 3-human		

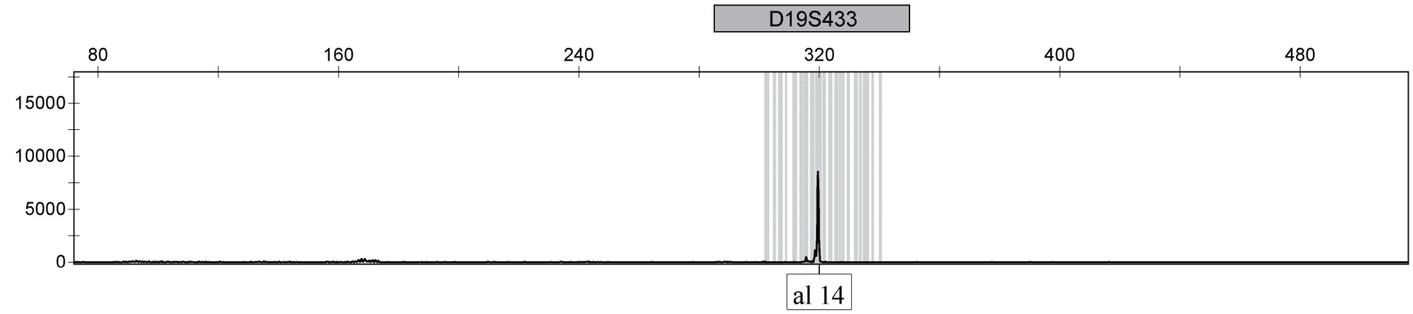

Sample File	Sample Name	Panel	OS	SQ
53_E07_CellLineAuthentication-0309.fsa	H1299	STR Profile 4-human		

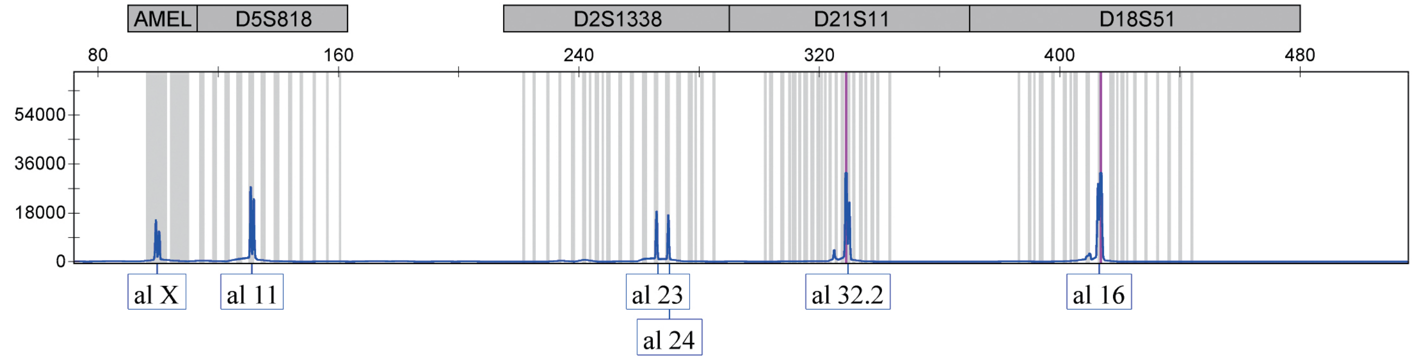

53_E07_CellLineAuthentication-0309.fsa	H1299	STR Profile 4-human		

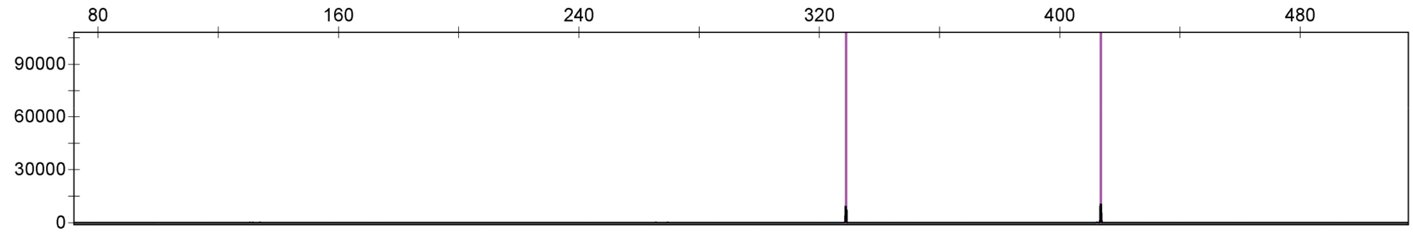

Supplement: Supplementary file 5 — Supplementary materials-NCI-H1299 STR profiling [file 41419_2022_5140_MOESM5_ESM.pdf]
